# Using Gene Editing Approaches to Fine-Tune the Immune System

**DOI:** 10.3389/fimmu.2020.570672

**Published:** 2020-09-29

**Authors:** Kristina Pavlovic, María Tristán-Manzano, Noelia Maldonado-Pérez, Marina Cortijo-Gutierrez, Sabina Sánchez-Hernández, Pedro Justicia-Lirio, M. Dolores Carmona, Concha Herrera, Francisco Martin, Karim Benabdellah

**Affiliations:** ^1^Genomic Medicine Department, GENYO, Centre for Genomics and Oncological Research, Pfizer-University of Granada (Andalusian Regional Government), Health Sciences Technology Park, Granada, Spain; ^2^Maimonides Institute of Biomedical Research in Cordoba (IMIBIC), Cellular Therapy Unit, Reina Sofia University Hospital, University of Cordoba, Cordoba, Spain; ^3^LentiStem Biotech, GENYO, Centre for Genomics and Oncological Research, Pfizer-University of Granada (Andalusian Regional Government), Health Sciences Technology Park, Granada, Spain; ^4^Department of Hematology, Reina Sofía University Hospital, Córdoba, Spain

**Keywords:** immunotherapy, CARs, gene editing, graft-vs-host disease, base editors

## Abstract

Genome editing technologies not only provide unprecedented opportunities to study basic cellular system functionality but also improve the outcomes of several clinical applications. In this review, we analyze various gene editing techniques used to fine-tune immune systems from a basic research and clinical perspective. We discuss recent advances in the development of programmable nucleases, such as zinc-finger nucleases (ZFNs), transcription activator-like effector nucleases (TALENs), and clustered regularly interspaced short palindromic repeat (CRISPR)-Cas-associated nucleases. We also discuss the use of programmable nucleases and their derivative reagents such as base editing tools to engineer immune cells via gene disruption, insertion, and rewriting of T cells and other immune components, such natural killers (NKs) and hematopoietic stem and progenitor cells (HSPCs). In addition, with regard to chimeric antigen receptors (CARs), we describe how different gene editing tools enable healthy donor cells to be used in CAR T therapy instead of autologous cells without risking graft-versus-host disease or rejection, leading to reduced adoptive cell therapy costs and instant treatment availability for patients. We pay particular attention to the delivery of therapeutic transgenes, such as CARs, to endogenous loci which prevents collateral damage and increases therapeutic effectiveness. Finally, we review creative innovations, including immune system repurposing, that facilitate safe and efficient genome surgery within the framework of clinical cancer immunotherapies.

## Gene Editing Tools: An Update

The well-established field of genome editing (GE) facilitates precise genomic modifications to enable genetic diseases to be studied and treated. The precise modifications induced by GE tools generate small (1–10 bp) and large (up to 20 kb) changes using different strategies. These technologies can be classified into two main groups: (1) traditional approaches that generate double-stranded breaks (DSBs) in DNA at the desired genomic loci followed or not by the introduction of exogenous DNA; and (2) approaches enabling the genome to be modified without requiring double-stranded DNA (dsDNA) cleavage either by introducing small molecules forming a triplex structure or by combining deaminase enzymatic activity with specific impaired catalytic endonucleases. In this review, we evaluate the different techniques used to engineer immune cells in the treatment of primary immunodeficiencies and acquired diseases such as cancer and infectious diseases.

### DSB-Based Gene Editing Approach

Over the last three decades, the following major specific endonucleases (SENs) have been successfully developed for both basic research and clinical purposes: meganucleases (MGNs), transcription activator-like effector nucleases (TALENs), megaTAL nucleases, ZFNs and, more recently, clustered regularly interspaced short palindromic repeat (CRISPR)-Cas-associated nucleases. The success of SENs is evidenced by the 43 on-going clinical trials using ZFNs (14), CRISPR/Cas9 nucleases (23), and TALENs (6) to treat infectious diseases (HIV-1, HPV), cancer, as well as blood and metabolic disorders (Clinicaltrials.gov June 2020). This field began to develop in 1994 when Dr. Maria Jasin and her team discovered that the generation of double-stranded breaks (DSBs) in mammalian DNA favors homologous recombination (HR) repair and that DSBs can be directly repaired by non-homologous end joining (NHEJ) (1–3). These discoveries laid the foundations of SEN-based gene editing research (4). The first SENs used to create specific DSBs for genome editing were meganucleases (MGNs), a group of endonucleases that recognize 12–45 bp DNA sequences (5–9). More versatile GE tools were developed some time later, with the use of FokI catalytic and zinc-finger domains to generate the first ZFNs (10). Soon afterward, TALENs were designed based on the bacterial system of TAL effectors (TALEs). TALENs have two different domains: a DNA-binding domain, the TALE proteins, that can be designed to bind the desired sequence (10), and a Fok1 endonuclease domain (11). With its simple design, the CRISPR/Cas system, the most versatile gene editing tool, is derived from bacteria, particularly from an adaptive immune system found in prokaryotes, which provides defense against viral infections and plasmids. CRISPR/Cas proteins form a complex with RNA molecules which guides Cas endonucleases to the target DNA to be cleaved (12). RNA nucleotides are the only part of the system that needs to be changed to specifically cut a new target site in the genome (13, 14).

### Programmable Editing Without Double-Stranded DNA Cleavage

**FIGURE 1:**
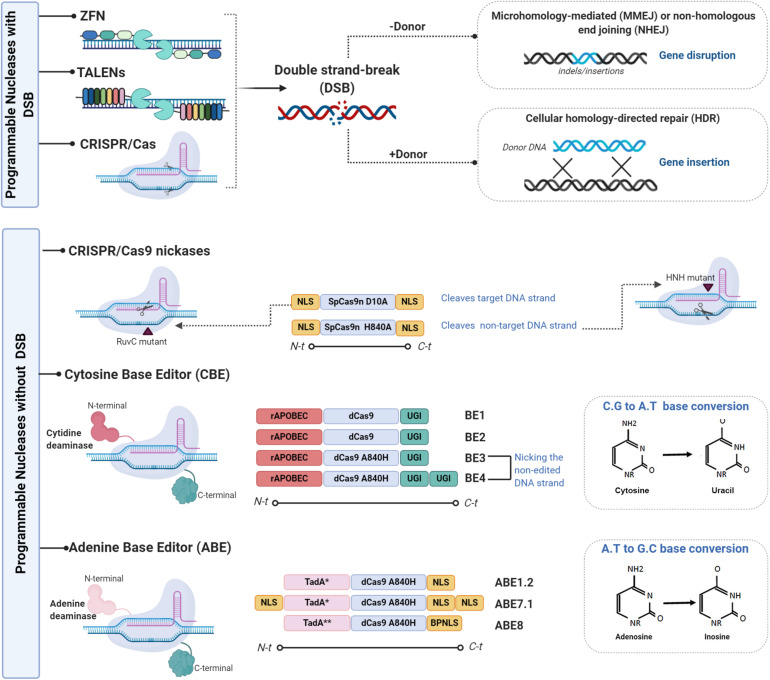
Representative scheme of the different genome editing tools used to improve the immune system. There are two variants of genome editing technologies: those that introduce double-stranded breaks (DSBs) into DNA and those that enable genome editing without DSBs. The first variant is mainly composed of the components ZFNs, TALEN, and CRISPR/CAS which are used to enhance immune system capacity. ZFNs are chimeric proteins containing a DNA binding domain (3–5 zinc-finger domains) and a Fok1 endonuclease domain (114). Each zinc-finger domain is specifically designed to bind to virtually any DNA sequence. ZFN cleavage activity needs to be dimerized given that Fok1 acts as a dimer. Two ZFNs therefore need to be designed, each targeting a DNA sequence separated by a short sequence from the recognition site of the other ZFNs in a head-to-head fashion. As ZFNs, TALENs contain two different domains: the DNA binding domain of the TALE protein designed to bind the desired sequence (10) and the Fok1 endonuclease domain (11). As TALENs only act as dimers, two TALENs must be designed to bind to the target locus in a face-to-face fashion to cleave the target sequence (110). CRISPR/Cas, the last described SENs, is the easiest to design and the most versatile gene editing tool. It is derived from the adaptive immune system of prokaryotes, which provides a defense mechanism against certain viral infections and plasmids. The CRISPR/Cas protein forms a complex with the RNA molecules crRNA (CRISPR RNA) and crRNA (tracrRNA) which guides the Cas protein to the target DNA and produces the cleavage (12). The only part that needs to be changed to specifically cut a new target site in the genome is 20 crRNA nucleotides (13, 14). Various modifications to the gRNA design and to the Cas9 protein have been made to reduce off-target activity. A mutated Cas9 nickase has been generated to expand the CRISPR genome editing system (111). The second group is mainly composed of CRISPR-CAS nickases variants and of two base editor (BE) variants, the cytosine base editor (CBE) and the adenine base editor (ABE). The first variant is based on simple APOBEC deaminase system named BE1, which fuses APOBEC1 and dead Cas9 from *Streptococcus pyogenes* with D10A and H840A mutations (28). Its lower efficiency is attributable to uracil DNA glycosylase (UDG) which catalyzes the removal of U from DNA in cells and initiates base-excision repair (BER), thus converting the U:G pair to the C:G pair (115). The uracil-DNA glycosylase inhibitor (UGI) was fused to the C terminus of BE1 to create the second-generation BE2 system, with an improved base editing yield of 50%. A further improvement was implemented for the third-generation BE3 system. The improved BE4 base editor contains a rAPOBEC cas9 linker expanded to 32 amino acids, a Cas9n-UGI linker expanded to 9 amino acids, as well as the addition of a second copy of UGI to the C terminus of the constructs (116); BE3 and BE4 have been validated for use as base editors of human primary T cells (55). The replacement of the APOBEC1 component in BE3 with natural adenine deaminase *Escherichia coli* TedA led to the creation of the first adenine base editor ABE which was followed by ABE1.2 After several target mutations and optimizations, the ABE7.1 base editor was released. This was followed by the latest version ABE8 with its base editing facility particularly for HSPCs and human primary T cells (33). Figures were created by BioRender.com.

As mentioned earlier, the introduction of DSBs by SENs, followed by the activation of cellular repair machinery, can generate unwanted side effects such as off-target site indels, large deletions, and translocations. In addition, multiplex editing can lead to relatively frequent translocations and/or chromosomal rearrangements (15, 16). The following alternative systems can be devised to edit the genome without generating DSBs (Figure 1): (1) viral episomal vectors, such as adeno-associated viruses (AAVs), containing donor DNA (17–19); (2) triplex-forming oligonucleotides (TFO), which are able to deform DNA strands, triggering repair mechanisms without inducing DNA breaks (20–22); (3) mutated Cas9, where the RuvC nuclease domain that targets the non-complementary DNA strands and the HNH nuclease domain that targets the complementary strand are mutated converting Cas9 into a DNA nickase (23, 24); and (4) base editing (BE), a genome editing method that generates exact point mutation in both genomic and RNA sequences without DSBs (25). Two different base editors, the cytosine base editor (CBE) and the adenine base editor (ABE), have been developed over the last 3 years. The CBE, based on cytidine deamination, a common natural DNA and RNA modification, is involved in several normal biological processes (26). The principal deamination enzymes are the apolipoprotein B mRNA editing enzyme, a catalytic polypeptide-like 3 G (APOBEC), and the activation-induced cytidine deaminase (AID) enzyme (27). These enzymes can be combined with the versatile CRISPR/Cas system for genome base editing. A substantial number of studies have been published on improvements made in areas such as specificity and efficacy (28). *Petromyzon marinus* cytidine deaminase 1 (PmCDA1), identified in the lamprey genome, was used to increase BE versatility (29). The substitution of SpCas9 by *Staphylococcus aureus* (SaCas9) or *Lachnospiraceae bacterium* Cpf1 (Cas12) facilitates base editing in AT-rich organisms and interrogation of more genomic loci (30). Unlike the CBE, ABEs have no natural eukaryotic adenosine deaminase enzymes capable of acting on single-stranded DNA (ssDNA). The first ABEs were developed using adenine deaminase from *Escherichia coli* TedA (31). Over the last 2 years, several modifications to evolving ABE variants have been made: modified nuclear localization signals (NLSs) and codon usage, inclusion of ancestral deaminases resulting in BE4max, AncBE4max, and ABE max, as well as ABE8s with no significant levels of off-target adenine deamination in genomic DNA (32, 33).

Regardless of the system used, given that GE technologies need to be sufficiently specific for use in clinical treatments, the specificity of any proposed technique needs to be accurately assessed (34). Two articles in a recent issue of *Science* highlight the high rates of off-target mutation associated with base editing in two disparate rice and mouse organisms (35, 36). Therefore, to be used in clinical settings, improvements have been made to increase BE specificity. Both CBEs and ABEs have been optimized, resulting in BE variants (Figure 1) with improved efficacy and specificity (32, 37, 38). Finally, the more powerful and precise genome editing technique, prime editing (PE), has an impressive array of applications. PE involves the fusion of two proteins from a Cas9 nickase domain and an engineered reverse transcriptase domain (39, 40). The possibility of introducing a point mutation at a specific location offers greater targeting potential than other SENs, especially with regard to non-dividing cells such as those in the nervous system (41).

## Advances in Genetic Engineering of Immune Cells

**FIGURE 2:**
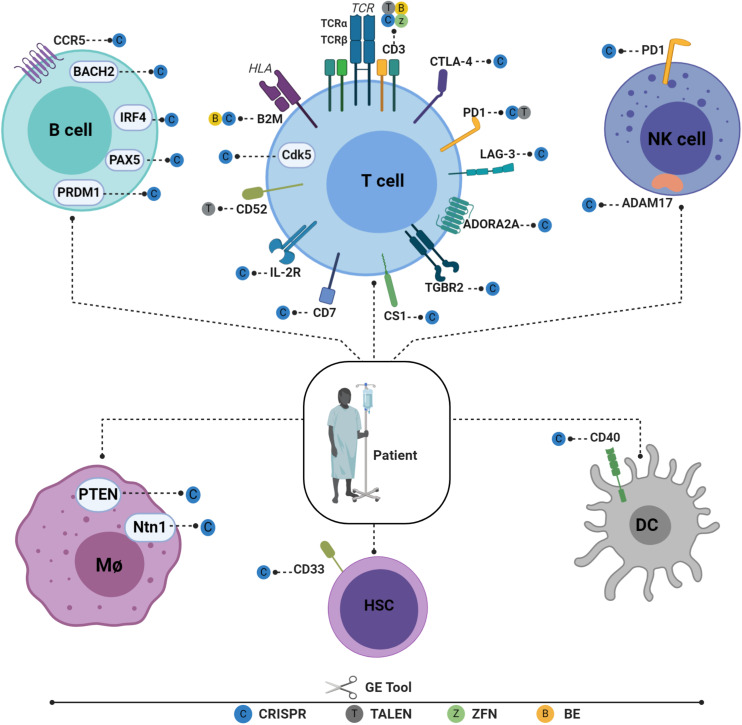
Recent advances in engineering different immune cell types for immunotherapy applications. Engineered T cells in the B2M gene have lowered HLA class I antigen expression on the cell surface and have reduced the possibility of graft rejection. TCR/CD3 cells have been knocked out to reduce GVHD and to enable physiological CAR expression, thus enhancing CAR T potency. Tumor-suppressive microenvironments have been overcome by downregulating CTLA-4, PD1, and LAG-3. T cells have also been engineered to ignore suppressive signals by expressing dominant negative TGF beta receptors (TGBR2). On the other hand, to engraft T cells under lymphodepleting preparative conditions, the elimination of CD52 is required to enable T cells to resist alemtuzumab-mediated lymphodepletion. Targeting CD7 prevents fratricide and enables the expansion of CD7 CAR T cells without compromising their cytotoxic function. IL-2R has also been engineered to facilitate IL12P70 expression in a controlled manner. Cyclin-dependent kinase 5 (*Cdk5*), a serine/threonine kinase, whose inhibition confers antitumor immunity, has been identified to regulate the PD-1/PD-L1 pathway. HSPCs are also gene edited to enhance adoptive immunotherapy. CD33-deficient human HSPCs resistant to CD33-targeted approaches have been produced to mitigate CART33 toxicity, to maintain myelopoiesis, and to prevent on-target off-tumor toxicity. Immune NK cells play an important role in host immunity against cancer and viral infections. Despite the low efficiency of viral and non-viral delivery methods, several NK cells can be edited to enhance their persistence, cytotoxicity, and tumor targeting (117). Dendritic cells (DCs) play a critical role in T-cell response instructions, with triple knockout established as proof of concept (118). A similar approach is used to target the costimulatory molecule CD40, whose disruption significantly inhibits T-cell activation, thus reducing graft damage and prolonging graft survival (88). Macrophages can also be edited using CRISPR-CAS9 by targeting USP7 and USP47, two genes that regulate inflammasome activation (119). The Ntn1 gene, thought to be involved in cell migration disruption, can also be targeted *in vivo* using nanoparticles encapsulating CRISPR/Cas9 RNPs under the control of the CD68 promoter (89). To elucidate its role in inflammation, the NEU1 gene can be targeted in macrophages using CRISPR-CAS9, thus demonstrating the role of NEU1 macrophages as inflammation enhancers (120). It is also possible to engineer B cells to express mature broadly neutralizing bNAb antibodies targeting IgH loci or safe harbor CCR 5 in the case of FIX. Figures were created by BioRender.com.

The remarkable progress made in GE tools in recent years has made it possible to engineer different immune cell types for use in immunotherapy (Figure 2). Cells previously considered highly resistant to genetic modification can now be gene-edited very efficiently using these new technologies. This enables the behavior of different immune cells to be fine-tuned by deleting or enhancing endogenous gene expression and by inserting new genes in safe harbor loci. HSPCs, T cells, B cells, macrophages, natural killers (NKs), and dendritic cells (DCs) can now be efficiently manipulated to boost their potency (Figure 2). By editing different immune cell types, a specific cellular circuit can be shut down or specific endogenous immune pathways repurposed for new functions. Below, we review recent advances in GE methods that have been successfully applied to different immune cell types.

### Hematopoietic Progenitor and Stem Cells

Hematopoietic progenitor and stem cells (HPSCs), some of the most desirable target cells in adoptive immunotherapy, either by the introduction of genes encoding T-cell receptors (TCRs) and chimeric antigen receptors (CARs) that target tumor-associated antigens. Unlike other hematopoietic cells, HPSCs, with their long-term engraftment capability, could provide a sustained source of effector cells. TCRs and CAR-HPSCs, which constantly produce T-lymphocyte progenitors, potentially increase the development of immunological memory. However, the engineering of HPSCs with CARs has two major drawbacks: (1) the increased likelihood, as compared with T cells, of insertional oncogenesis observed in previous clinical trials (42, 43). This can be countered by targeting CAR and TCR constructs at specific loci to raise physiological gene expression levels and to reduce the risk of insertional mutation; (2) as with other CAR strategies, the absence of cancer cell surface markers which increases on-target off-tumor toxicity. For example, the targeting of CD123 and CD33 in myelodysplastic syndrome (MDS) and in acute myeloid leukemia (AML) patients, respectively, results in toxicity due to the elimination of normal myeloid cells. MDS belongs to a group of heterogeneous diseases which is induced by defective hematopoiesis and characterized by bone marrow dysplasia and cytopenia. CAR T-cell therapy can be used to treat high-risk MDS patients, using CD123 to delineate malignant stem cell markers. Nevertheless, despite its upregulation in MDS stem cells, many studies have shown that CD123 is expressed in subpopulations of healthy non-malignant HSPCs (44). Thus, therapies, including CAR T therapy, envisaged for targeting CD123, need to take into account the on-target off-tumor effect (45, 46). Clinical trials have begun on the treatment of blastic plasmacytoid dendritic cell neoplasm (BPDCN) (NCT03203369) and relapsed AML (NCT03190278) using universal T-cell targeting of CD123. Thus, to reduce these undesirable effects, effective complementary gene editing techniques include targeted removal and reductions to below the CAR T activation threshold, while maintaining normal CD123 expression in donor HSPCs. The myeloid differentiation cell surface marker CD33 can be targeted in CAR T AML therapies. This immune-targeting approach has been designed to target CD33 which is a myeloid differentiation antigen predominantly expressed on leukemic blasts in most AML patients (85–90%). However, this marker is not only present on leukemic cell surfaces but also in healthy cells, as clearly evidenced by the withdrawal from the market in 2010 of gemtuzumab, a conjugated anti-CD33-antibody, due to bone marrow toxicity. CD33-deficient human HSPCs resistant to CD33-targeted therapy mitigate CART33 toxicity to sustain myelopoiesis and to prevent on-target off-tumor toxicity (47). AML and MDS strategies can also be used for other cell-surface antigens in CAR settings and in all antigen-specific immunotherapies.

### B Cells

B cells are key regulators of humoral responses in adaptive immune system. Mature B cells, also called plasma cells, which reside in bone marrow, are in charge of producing and secreting antibodies. Most gene editing of B cells relies on the development of cellular humoral vaccines, thus avoiding the need for repetitive administration of antibodies (48). The replacement of heavy and light chain B-cell receptors with sequences encoding a suitable monoclonal antibody in an allergenic manner could improve immunization (49, 50). Based on this hypothesis, GE could be used to express recombinant antibodies under the control of endogenous regulatory elements, enabling strict regulation of gene expression in response to specific antigens. This would facilitate the production of appropriate concentrations of the corresponding antibodies (49). Genome editing of human B cells mediated by homology-driven repair (HDR)/CAS9 has also been used to produce anti-HIV-1 broadly neutralizing antibodies (bNAbs) (51) and to boost the capacity of long-lived plasma cells to physiologically produce proteins with therapeutic applications (30). Hung et al. have generated active FIX-secreting human plasma cells at the CCR5 safe harbor locus using the HDR-mediated method. The engineered B cells expressed the active recombinant FIX gene, demonstrating the possibility of editing primary B cells with high specificity and efficacy. This approach paved the way for future clinical applications involving primary B cells (30). Cas9-mediated disruption has also been tested on naïve B cells for IRF4, PRDM1, PAX5, and BACH2 genes, with over 80% efficacy, thus proving the feasibility of gene knockdown in this cell type and its potential use in immunotherapeutic treatments (30).

### T Cells

Given their central role in immune responses, primary human T cells have great potential for use in immune cell gene therapies. Genetically modified T cells have been investigated for many years in adoptive cellular immunotherapies for the treatment of cancer, infectious diseases, and immunodeficiencies. However, although GE technologies were initially successful in other cell types, their use in primary human T cells was hampered by low-efficiency delivery methods and the vulnerability of these cells to physical and chemical challenges. Nevertheless, as mentioned earlier, new developments have led to increased efficiency and reduced damage overall, specifically in T cells. T cells from different sources, such as peripheral blood, tumors, and bone marrow, with different phenotypes, including αβ, Ɣδ, and NKT, have been successfully edited and used in various immunotherapeutic approaches (see next section). Almost all the aforementioned GE tools have been applied to T cells for different purposes. However, target genes such as programmed cell death-1 (PD1), TCRs, human leukocyte antigens (HLAs), and β2-microglobulin (β2M) have been used in multiple studies given their potential to increase or to control T-cell activity. It is important to note that both GE strategies, SENs and BEs, are now reaching efficacies over 80% of the desired modifications without a significant effect on T-cell physiology other than the desire. As with other cell types, two principal approaches, gene silencing and gene insertion, are used to fine-tune T-cell activity.

#### Gene Expression Silencing, Aimed at Blocking Gene Expression, Which Blocks T-Cell Activity or Interferes With Its Survival in the Host

##### (i) Programmed cell death-1

T cells, including tumor-infiltrating lymphocytes, were successfully gene edited using the three specific endonucleases (SENs), as well as the BEs described earlier, to target programmed cell death-1 (PD-1). By the electroporation with a clinical-grade mRNA encoding for ZFNs specific for PD-1, Bean et al. achieve an average modification frequency of 74% bulk population reducing to a minimal level the PD1 surface expression. Importantly, the authors do not report any adverse effects affecting T-cell functionality or phenotype (52). Other approaches based on TALEN and CRISPR/Cas9 methodologies were also considered. TALEN-mediated PD-1 gene inactivation in melanoma CD8^++^ T cells was triggered using an optimized mRNA-electroporation protocol (53, 54). More recent ABE- and CEB-based approaches were also considered to significantly reduce the risks associated with unintended genomic alterations and genotoxicity (33, 55).

##### (ii) Human leukocyte antigen

Human leukocyte antigen (HLA) expression on CAR T cells can lead to immediate rejection by the host recipient through recognition of non-self HLAs. GE-based methods are used to eliminate HLA molecules from T-cell surfaces. As with PD-1, ZFNs (56), and the CRISPR system, base editing and mRNA electroporation have been used to eliminate HLA expression (9, 33, 55, 57, 58). Another possible strategy to eradicate HLA expression is the elimination of b2-microglobulin (B2M). HLA proteins are covalently associated with B2M in the endoplasmic reticulum, an association which is crucial for the formation and trafficking of functional HLA molecules on cell surfaces. However, some concerns have been raised with regard to the possible targeting of these HLA class I negative cells by NK cells (59, 60).

##### (iii) T-cell receptor

As discussed in the clinical applications section, TCR knockout has profoundly revolutionized immunotherapy. Genome editing of this locus has, on the one hand, consolidated the off-the-shelf nature of CAR T therapy by reducing graft-versus-host disease (GVHD) toxicity and has, on the other hand, facilitated the physiological regulation of CAR function and enhanced anti-tumor activity. The study by Berdien et al. was the first to completely shut down endogenous TCRs through the transfection of TALEN pairs specific to human TCR α and β chains (61). As noted later, TCR KO has become a well-established feature of clinical treatments (62–64).

##### (iv) Transformation of growth factor beta receptor 2 (TGFBR2) proteins

Another major issue is the complexity of the tumor microenvironment (TME) which inhibits CAR T therapies. One of the most important regulators in the TME is TGF-β. There are three TFG-β ligands, TGF-β1, TGF-β2, and TGF-β3. The receptor most commonly upregulated in tumor cells is TGF-β1, which negatively regulates CAR T-cell cytotoxic function via TGF-β receptors. With an attempt to enhance CAR T cells using CRISPR/Cas9, Tang et al. managed to knock down TGFBR2 and to improve the *in vivo* CAR T cells in an animal model (65).

##### (v) Cyclin-dependent kinase 5 (*Cdk5*)

The serine/threonine kinase Cdk5 has been identified to regulate the PD-1/PD-L1 pathway. Its inhibition confers antitumor immunity due to interference with interferon regulatory factor 2 (IRF2) and interferon regulatory factor-binding protein 2 (IRF2BP2) (66, 67). Consistent with this model, the depletion of CDK5 by shRNA leads to the hyperphosphorylation of IRFBP2, increased IRF2 expression, and lower PD-L1 levels (68, 69). In addition, CRISPR-mediated disruption of CDK5 in cancer cells results in suppression of tumor growth (69, 70).

##### (vi) Multiplex gene knockout

Targeting two or more loci simultaneously is a pre-requisite for certain therapeutic approaches (71). For example, the CD52 locus targeted by alemtuzumab, along with the TCR and PD1 loci, can be targeted simultaneously to facilitate engraftment of CAR T cells resistant to alemtuzumab and PD-L1. CD52/TCR KO T cells were successfully obtained by TALEN mRNA electroporation (72), and this approach has recently been used in clinical therapies (73). Similarly, TCR/PD1 KO T cells were obtained using highly specific non-conventional TALEN technology for multiplex genome editing (74). The Ren research group carried out a quadruple gene ablation using CRISPR system to generate dual inhibitory resistant universal CAR T cells deficient in TCR, HLA-I, PD-1, and CTLA-4. The multiplex genome editing of CAR T cells with the one-shot CRISPR system was found to be highly efficient (57, 58). Despite these impressive results, undesirable effects, such as chromosomal re-arrangement and translocation, are highly possible. However, both genome base editing and the nickase variant of the CRISPR system enable several genes to be targeted simultaneously without any adverse effects. This multiplex approach using BE tools has been used successfully to target TCR, B2M, and PD-1 genes in a single round of electroporation. All these genes were silenced without DSBs while preserving their capacity to mediate target cell killing (33, 55).

#### Insertion of New Genes Into Desired Loci: Dual Knockin Effect

Recent advances in gene editing technologies have enabled the knockout of endogenous genes and at the same time the introduction of large DNA sequences into specific loci to integrate new genetic instructions into specific endogenous loci to modulate T-cell function and specificity. These strategies were successfully adapted to CAR T therapy, simultaneously knocking out one gene and expressing another. This approach takes advantage of the tightly regulated nature of immune pathways, enabling the endogenous genes to be precisely repurposed. In this regard, Roth et al. reported a virtual total loss of endogenous TCRs which were replaced by specific tumor-associated antigen TCRs using CRISPR/Cas9 ribonucleoproteins (RNPs) co-transfected with dsDNA HDR templates (75). With the aid of this feature, Eyquem et al. developed a similar strategy to incorporate the CAR construct into the TRAC locus (76). As part of a more sophisticated approach, Sachdeva et al. used the signaling pathways of TCR, CD25, and PD1, three major players involved in T-cell activation, to express CAR and IL12-P70 genes in a controlled manner (77). The highly regulated nature of immune pathways enables therapeutic genes to be expressed in a tumor cell–dependent manner, thus reducing several side effects associated with uncontrolled continuous expression (76, 77).

### Natural Killer Cells

Natural killer (NK) cell lymphocytes play an important role in the innate immune system due to their ability to kill a variety of target cells, including cancer cells, and can rapidly respond in a thymus-independent manner without previous recognition of the antigen (78). Their effector capacity depends on the balance between activating and inhibiting signals triggered by specific receptors that recognize ligands on stressed cells such as tumoral and infected cells (79). Recent studies show that NK cells can be genetically engineered to express chimeric antigen receptors (CARs) (80–82). However, few studies have reported gene editing modifications in NK cells due to their low rates of transduction with viral systems and the difficulty of NK expansion *in vitro* (83, 84). These issues are well illustrated by the first study to demonstrate efficient HDR genome editing in NK cells which appeared in 2020 (84). Another study, using the CRISPR/Cas9 RNP system, showed that NHEJ-GE KO of ADAM17 and PDCD1 (85) induces enhanced killer phenotypes via ADCC and non-ADCC pathways, respectively.

### Dendritic Cells

Dendritic cells (DCs), considered to be at the center of the immune system, provide a crucial link between innate and adaptive immune responses. These cells, first described by Steinman and Cohn, have a unique immunomodulatory capacity. The few studies in the literature on dendritic cell editing mainly focus on graft rejection mediated by activated T cells. CD40, a key molecule involved in DC activation and maturation, is deeply implicated in communication between DCs and T cells. This interaction relies on antigen presentation and subsequent helper and cytotoxic T-cell priming (86, 87). Thus, any CD40 reprogramming of DCs will allow transplant tolerance, and with this in mind, Zang et al. used a novel delivery method consisting of poly(ethylene glycol)-*block*-poly(lactide-*co*-glycolide) (PEG-*b*-PLGA)-based cationic lipid-assisted nanoparticles (CLANs). This method is capable of delivering of delivering Cas9 mRNA (mCas9) and a guide RNA targeting the costimulatory molecule CD40 (gCD40) both *in vivo* and *in vitro*. CD40 knockdown significantly reduced T-cell activation, thus alleviating graft damage and prolonging graft survival (88).

### Macrophages

Macrophages and their monocyte precursors are members of the innate immune system and form part of the mononuclear phagocyte system. Macrophage gene editing is currently being tested for use in the therapeutic treatment of several diseases. Luo et al. have developed CRISPR/Cas9 vectors using a plasmid delivery system under the control of the CD68 promoter which is capable of boosting gene expression specifically in monocytes and macrophages. In addition, these plasmids were encapsulated in PEG-*b*-PLGA-based CLANs, which are redirected to B cells, neutrophils, monocytes, and macrophages (89). Luo et al. performed *in vivo* targeting of the *NTN1* gene, whose overexpression is associated with type 2 diabetes (T2D) and monocyte/macrophage-specific expression, with no off-target effects and an editing efficiency of 10.1% at a dose of 1 mg/kg and of 19.6% at 2 mg/kg as compared with 1.2 and 2.3% for indels detected in neutrophils, respectively (89). Macrophages were also genetically edited for cell-based cancer immunotherapy through the elimination of CD47:SIRP-α interactions by knocking out SIRP-α using the CRISPR-Cas9E20 system and by introducing an E20 tag at the protein N terminus, thus facilitating self-assembly with arginine-coated gold nanoparticles (ArgNPs). This novel complementary strategy, which enables over 30% of genes to be edited, provides a novel and complementary approach to cancer immunotherapy (90).

## Genome Editing Strategies to Improve Car T Cancer Immunotherapy

Cancer, which is a complex disease caused by a variety of genetic and epigenetic changes, is a major threat to human life and public health. Conventional strategies, such as surgery, radiotherapy, and chemotherapy, have made significant advances in the treatment of cancer. However, manipulation of the immune system has become one of the most promising therapeutic approaches. Among the vast repertoire of effective antitumoral molecules and drugs, genetically modified immune cells have emerged as a truly effective therapeutic approach. One of the most promising anticancer treatments is CAR T-cell therapy, which mainly involves a redirection of immune cells against tumors. Although CAR-based therapy has already proved its efficacy in certain hematological malignancies, several challenges remain to be addressed: (1) the biological characteristics of autologous T cells; (2) the expansion of CAR T cells and their potency; (3) the persistence of CAR T cells; (4) the determination of optimal CAR expression; (5) the determination of optimal T-cell subpopulations. In the following section, we describe different GE approaches for generating next-generation CAR T cells with improved characteristics, as well as the potential clinical applications of CAR T gene editing products.

### Off-the-Shelf Engineered T Cells

Autologous CAR T cells are ideal for adoptive T-cell therapies due to the absence of allogeneic reactions. However, several limitations are associated with autologous T cells, mainly related to the cost and failure of personalized production processes. Some patients may not be able to meet numbers and quality of T cells required for the production process. Thus, autologous T cells may not be effective in the case of intrinsic dysfunctions associated mainly with previous treatments. To avoid these drawbacks, various groups have been investigating the production of suitable CAR T cells generated from healthy donors. To generate the CAR T cells to be administered to allogeneic transplant patients, the issue of the T-cell alloreactivity levels that trigger allograft rejection and GVHD needs to be resolved. As discussed earlier, GE can be used to resolve these potential issues by eliminating TCRs to prevent GVHD and by eliminating MHC class I and/or MHC class II molecules to prevent allograft rejection. Since the first study in 2012 in which TCRs were deleted using ZFNs (56), different approaches have been used to eliminate endogenous TCRs alone or combined with other target molecules to improve T-cell properties. Poirot et al. applied multiplexed gene editing to primary T cells for the first time using TALENs which simultaneously knocked out TCR and CD52 molecules (72). CD52, a surface protein expressed on immune cells, is targeted for lymphodepletion to delay allograft rejection. These edited T cells did not cause GVHD *in vivo* and were resistant to the anti-CD52 monoclonal antibody alemtuzumab. When equipped with a CD19 CAR, T cells are capable of destroying CD19^+^ tumor cells in *in vitro* and *in vivo* experiments. A similar strategy is used for expressing BCMA CAR T cells in multiple myeloma treatments, leading to the generation of *in vivo* activity similar to that of their wild-type counterparts (91) in clinical trials (NCT04093596). Liu et al. generated *TRAC*, *B2M*, and *PD-1* triple knockout T cells using the CRISPR/Cas9 system. They compared how double (TRAC, B2M) and triple (TRAC, B2M, and PD-1) knockout affected CD19-CAR T-cell activity, demonstrating *in vitro* that the triple CART showed higher efficiency than double knockout cells (92). As clinical proof of concept, TALEN-mediated TRAC/CD52 KO CD19 CAR T cells (UCART19) were administered to two infant patients with relapsed acute lymphoblastic leukemia (ALL), both of whom presented tumor remission, which was followed by successful allogeneic stem cell transplantation (allo-SCT), with no significant GVHD observed (73). Three multicenter clinical trials are ongoing on the safety and efficacy of UCAR19 cells in children with ALL (NCT02808442, pediatric ALL), adults with ALL (NCT02746952, CALM), and both age groups with lymphoid malignancies (NCT02735083). Other clinical trials have been initiated using universal CD123 CAR T cells generated by TALEN gene editing for BPDCN (NCT03203369) and relapsed AML (NCT03190278). The following clinical trials using CRISPR-edited T cells are also ongoing: TRAC/B2M KO CD19 CAR T cells for B-cell leukemia and lymphoma (NCT03166878; NCT03229876); TRAC/PD1 KO mesothelin CAR T cells for solid tumors (NCT03545815); TRAC KO dual CD19/CD22 and CD19/CD20 CAR T cells for B-cell leukemia (NCT03398967); CD19 TCR-CAR T cells for non-Hodgkin lymphoma and B-cell ALL using meganucleases (NCT03666000). However, as mentioned earlier, multiplex gene editing could involve chromosomal aberration and translocations. To prevent these major constraints, Webber et al. used CBEs to generate triple-negative CD19 CAR T cells (TRAC, B2M, PDCD1). These cells showed improved antitumoral capacity against target cells *in vitro* (55). It is important to note that the initial concerns regarding the off-target effects of BEs appear to dissipate with the development of new-generation BEs. The eighth generation of adenine base editors, ABE8, provides for multiplexed editing of B2M, CIITA, and TRAC genes, with an editing efficiency rate of over 98% (33). TCR gene editing also benefits immunotherapy approach based on cancer-specific TCR surface expression, in which the presence of both endogenous TCRs and cancer-specific CARs induces the formation of heterodimers which, in turn, reduces therapeutic effectiveness. Legut et al. therefore co-transduced primary T cells expressing cancer-specific αβ and γδ TCRs, with a lentiviral vector encoding a CRISPR/Cas9 system designed to target TRBC*1* and *TRBC2* genes. The response of the double-transduced T cells to target B-LCL and melanoma cell lines was more sensitive, strong, and efficient than that of standard TCR-transgenic T cells (63). These preliminary findings were clinically corroborated in a phase I clinical trial using autologous NY-ESO-1 TCR T cells after CRISPR-Cas9 editing of *TRAC*, *TRBC*, and *PDCD1* loci began (NCT03399448). Although the edited T cells in patients resisted for up to 9 months, only one patient experienced tumor regression (71). TCR genes were also knocked out to produce fratricide-resistant CD3-specific CAR T cells to treat T cell ALL, whereas TCR CD3^–^CAR T cells created by TALEN editing generated strong antileukemic effects on a xenograft mouse model (93). To treat T-cell malignancies, other markers such as CD7 were knocked out. Using the CRISPR/Cas9 toolkit, CD7 CAR T cells (UCART7) mediated the efficient destruction of malignant T cells with no significant T-cell fratricide reported (94–96).

### Expansion, Persistence, and Potency of CAR T Cells

Although CAR-modified T cells have been successfully used in the treatment of hematologic malignancies, several limitations still need to be resolved, especially with regard to solid tumors, which are mainly associated with an unfavorable TME, which includes the upregulation of inhibitor receptors (IRs), leading to the activation of intrinsic inhibitory pathways. Modulation of TME responses by antagonizing tumor-associated negative immune regulators such as PD1, TGF-β, and adenosine is considered a desirable treatment strategy. One of the most extensively characterized T-cell IRs is programmed cell death 1 (PD-1), also known as CD279, which is a cell surface immunoinhibitory receptor expressed by a wide range of immune cells such as T cells, B cells, dendritic cells (DCs), natural killers (NKs), and myeloid cells (97). Its activation depends on the PD-L1 and PD-L2 ligands present in tumor cells, whose interaction with PD-1 inhibits T-cell activation and proliferation by inducing energy and an immunosuppressive process in consonance with the TME (98). PD1 blockage is being tested as a novel immunotherapeutic target in different cancers. FDA-approved PD-1 and PD-L1 antibodies, such as nivolumab, pembrolizumab, and MPDL3280A, are indicated in the treatment of melanoma, metastatic bladder cancer, and glioblastoma (99–101), although PD-1 gene editing may increase CAR T potency. The GE tools described, including TALEN, CRISPR-Cas9, and ABE, have all been used to target *PD1* loci (54, 57, 58, 71, 102, 103). In all cases, the disruption of this protein enhances cellular immune responses, thus increasing cancer cytotoxicity and enhancing cancer immunotherapy. Clinical trials with PD-1 knockout autologous T cells are currently under way for the treatment of cancers such as prostate cancer (NCT02867345), bladder cancer (NCT02863913), and renal cell carcinoma (NCT02867332). Other clinical trials on where PD-1 genes are downregulated to fight lung cancer (NCT02793856), esophageal cancer (NCT03081715, phase II), and multiple tumors (NCT03545815, NCT03747965, NCT03399448) are ongoing. TGF-β (types 1, 2, and 3) is secreted by stromal cells such as cancer-associated fibroblasts, blood endothelial cells, mesenchymal stem cells, lymphatic epithelial cells, and pericytes. TGF-β, one of the most important immunosuppressive molecules in the TME, contributes to cancer initiation and progression. TGF-β favors the conversion of CD4^+^ effector T cells into CD4^+^ Tregs and blocks the secretion of cytotoxic molecules and cytokines in CD8^+^ T cells. In CAR T cells, the presence of TGF-β1 accelerates the exhaustion of CAR T cells by upregulating the PD1- and FOXP3-dependent Treg-like phenotype of CAR T cells (65). *TGFBR2* knockout enhances the antitumor efficacy of CAR T cells *in vivo* in cell line–derived xenograft and patient tumor–derived xenograft (PDX) mesothelin pancreatic carcinoma. After the PDX tumor was eliminated by *TGFBR2*-KO CAR T cells, the treated mice were re-challenged with novel contralaterally reinoculated patient-derived tumor cells, showing a persistence and antitumor properties (65). The adenosine A2A receptor, also known as ADORA2A, plays a regulatory role in the adaptive immune system. Adenosine, which is generated by tumor cells, strongly inhibits endogenous antitumor T-cell responses by activating ADORA2A. ADORA2A ablation could block adenosine signaling inhibition in T cells, thus providing a feasible way to study hostile TMEs, one of the major barriers to inhibiting immune reactions (104). This could be used to enhance CAR T-cell efficiency. Beavis et al. have highlighted the potential of targeting A2AR-mediated suppression to enhance CAR T-cell activity, particularly against solid tumors, which has, so far, been less impressive and in which adenosine-mediated immunosuppression is more prevalent due to the hypoxic environment (105). A2AR antagonists have undergone phase III clinical trials for Parkinson’s disease and are currently in phase I trials for oncology, indicating the highly translational nature of this approach (ClinicalTrials.gov, NCT02655822). A2AR was knocked down using retroviral shRNA to downregulate the target receptor and a second retroviral vector with an anti-HER2 CAR.

### Persistence of CAR T Cells

Robust *in vivo* expansion and persistence of genetically modified T cells are considered prerequisites for positive responses in hematologic malignancy patients. On the one hand, cytotoxic T lymphocyte-associated protein 4 (CTLA-4 or CD152) is a protein receptor expressed in activated T cells and regulatory T cells that bind to B7 ligands in antigen-presenting cells to transmit inhibitory signals to T cells following activation, downregulating IL-2 and reducing cell division. CTLA4 disruption with CRISPR/Cas9 increases TNF-α and IFN-γ secretion, leading to a significant increase in the apoptosis of human adenocarcinoma and bladder carcinoma tumor cells (106, 107). On the other hand, LAG-3, a major inhibitory receptor belonging to the IgG family, has 20% similarity with CD4 and binds to MHC class II receptors with higher affinities than CD4. LAG-3 expression occurs on activated CD4 and CD8 T cells, regulatory T cells (Tregs), NK cells, B cells, and plasmacytoid dendritic cells. LAG-3 is upregulated on exhausted T cells as compared with effector and memory T cells (108) in cancer, chronic infections, and autoimmunity (109). Blocking both PD1 and LAG-3 pathways with monoclonal antibodies synergistically reverses T-cell exhaustion (110) and increases memory T-cell formation (111). In addition, clinical trials blocking of LAG-3 alone or combined with anti-PD1 for the treatment of solid tumors are currently under way (NCT01968109). Recent clinical reports highlight the relationship between individual responses and the frequencies of infused CD8^+^ CTL19 cell expressing PD-1 TIM3 and LAG-3. Analysis of PD-1 co-expression with LAG3-3 and TIM-3 revealed that CD8^+^LAG3^+^ cells expressing PD1 are associated with poor responses, whereas patients in full remission were infused with products containing significantly lower frequencies of these cells (112). Although several preclinical studies have been carried out to evaluate the effect of lymphocyte activation gene 3 (LAG3) ablation, no definitive data are available on, for example, the bulk population of 70% of LAG-3 KO CAR T cells, which show no significant improvement, as compared with CAR T cells, with regard to efficiency or T-cell exhaustion in a xenograft CD19^+^Raji-NSG mouse model (113).

## Conclusion

The possibility of modifying the genome of human cells in general and immune system cells in particular opens up a whole range of opportunities hitherto unimaginable. The generation of large deletions/insertions, transcription factor targeting, and epigenetic markers have enabled scientists and clinicians to modulate gene expression with unprecedented precision. Despite some remaining limitations with respect to specificity and efficiency, various several studies carried out in this field have produced encouraging results and feasible clinical applications such as those utilizing available gene editing tools to generate off-the-shelf CAR T cells. A new system, in which endogenous TCR and HLA molecules on allogeneic T cells are eliminated, leading to a reduction in GVHD and rejection by the transplant recipient’s immune system, has also been developed.

These off-the-shelf–based therapies are of special interest to patients who, due to previous treatments or disease progression processes, have T cells deficient in either number or quality. Universal T cells can be generated and stored for later immediate use. GE approaches have also used immune checkpoint inhibitor therapies. Several immune checkpoint molecules expressed on immune cells have been successfully targeted using SENs to enhance the effect of CAR T cells, particularly CTLA-4, PD1, and LAG-3, with the elimination of PD1 most extensively studied in clinical settings. Blockage of the PDL1-PD1 axis enhances the cytotoxicity of T cells, an effect which is intensified when inhibitory checkpoints, such as CTLA-4, PD-1, and LAG-3, are simultaneously targeted. Although these strategies are being tested in several clinical trials, one of the major shortcomings of the multiplex strategy is associated with the simultaneous presence of multiple DSBs, which dramatically increases the likelihood of non-lethal but potentially toxic translocation. This problem is likely to be solved by the use of emerging efficient base-editing technologies to drastically reduce genotoxicity and to eliminate the possibility of genomic translocation. All these emerging technologies open up new possibilities and applications in the field of biomedicine. Although the results of early phases of clinical trials evolving these systems are encouraging, the limited number of patients involved hinders for the moment a definitive conclusion concerning the safety of those approaches.

## Author Contributions

KP, MT-M, NM-P, and MC-G: manuscript writing and figure processing. SS-H and PJ-L: manuscript writing. MDC and CH: manuscript review. FM and KB: manuscript writing and final approval of manuscript. All authors contributed to the article and approved the submitted version.

## Conflict of Interest

The authors declare that the research was conducted in the absence of any commercial or financial relationships that could be construed as a potential conflict of interest.

## References

[1] Rouet P Smih F Jasin M. Introduction of double-strand breaks into the genome of mouse cells by expression of a rare-cutting endonuclease. *Mol Cell Biol.* (1994) 14:8096–106. 10.1128/mcb.14.12.8096 7969147 PMC359348

[2] Rouet P Smih F Jasin M. Expression of a site-specific endonuclease stimulates homologous recombination in mammalian cells. *Proc Natl Acad Sci USA.* (1994) 91:6064–8. 10.1073/pnas.91.13.6064 8016116 PMC44138

[3] Smih F Rouet P Romanienko PJ Jasin M. Double-strand breaks at the target locus stimulate gene targeting in embryonic stem cells. *Nucleic Acids Res.* (1995) 23:5012–9. 10.1093/nar/23.24.5012 8559659 PMC307507

[4] Porteus MH. A new class of medicines through DNA editing. *N Engl J Med.* (2019) 380:947–59. 10.1056/nejmra1800729 30855744

[5] Silva G Poirot L Galetto R Smith J Montoya G Duchateau P Meganucleases and other tools for targeted genome engineering: perspectives and challenges for gene therapy. *Curr Gene Ther.* (2010) 11:11–27. 10.2174/156652311794520111 21182466 PMC3267165

[6] Galetto R Duchateau P Paques F. Targeted approaches for gene therapy and the emergence of engineered meganucleases. *Expert Opin Biol Ther.* (2009) 9:1289–303. 10.1517/14712590903213669 19689185

[7] Redel BK Prather RS. Meganucleases revolutionize the production of genetically engineered pigs for the study of human diseases. *Toxicol Pathol.* (2015) 44:428–33. 10.1177/0192623315613160 26516165 PMC4805444

[8] Paques F Duchateau P. Meganucleases and DNA double-strand break-induced recombination: perspectives for gene therapy. *Curr Gene Ther.* (2007) 7:49–66. 10.2174/156652307779940216 17305528

[9] Fajardo-Sanchez E Stricher F Paques F Isalan M Serrano L. Computer design of obligate heterodimer meganucleases allows efficient cutting of custom DNA sequences. *Nucleic Acids Res.* (2008) 36:2163–73. 10.1093/nar/gkn059 18276641 PMC2367722

[10] Kim YG Cha J Chandrasegaran S. Hybrid restriction enzymes: zinc finger fusions to Fok I cleavage domain. *Proc Natl Acad Sci USA.* (1996) 93:1156–60. 10.1073/pnas.93.3.1156 8577732 PMC40048

[11] Wei C Liu J Yu Z Zhang B Gao G Jiao R. TALEN or Cas9 – rapid, efficient and specific choices for genome modifications. *J Genet. Genomics.* (2013) 40:281–9. 10.1016/j.jgg.2013.03.013 23790627

[12] Chira S Gulei D Hajitou A Zimta AA Cordelier P Berindan-Neagoe I. CRISPR/Cas9: transcending the reality of genome editing. *Mol Ther Nucleic Acids.* (2017) 7:211–22. 10.1016/j.omtn.2017.04.001 28624197 PMC5415201

[13] Gasiunas G Siksnys V. RNA-dependent DNA endonuclease Cas9 of the CRISPR system: holy grail of genome editing? *Trends Microbiol.* (2013) 21:562–7. 10.1016/j.tim.2013.09.001 24095303

[14] Jinek M Chylinski K Fonfara I Hauer M Doudna JA Charpentier E. A programmable dual-RNA-guided DNA endonuclease in adaptive bacterial immunity. *Science.* (2012) 337:816–21. 10.1126/science.1225829 22745249 PMC6286148

[15] Cullot G Boutin J Toutain J Prat F Pennamen P Rooryck C CRISPR-Cas9 genome editing induces megabase-scale chromosomal truncations. *Nat Commun.* (2018) 10:1136. 10.1038/s41467-019-09006-2 30850590 PMC6408493

[16] Porteus M. Genome editing: a new approach to human therapeutics. *Annu Rev Pharmacol Toxicol.* (2015) 56:163–90. 10.1146/annurev-pharmtox-010814-124454 26566154

[17] Yin H Kauffman KJ Anderson DG. Delivery technologies for genome editing. *Nat Rev Drug Discov.* (2017) 16:387–99. 10.1038/nrd.2016.280 28337020

[18] Ehrhardt A Haase R Schepers A Deutsch MJ Lipps HJ Baiker A. Episomal vectors for gene therapy. *Curr Gene Ther.* (2008) 8:147–61. 10.2174/156652308784746440 18537590

[19] Miller DG Wang PR Petek LM Hirata RK Sands MS Russell DW. Gene targeting in vivo by adeno-associated virus vectors. *Nat Biotechnol.* (2006) 24:1022–6. 10.1038/nbt1231 16878127

[20] Chin JY Glazer PM. Repair of DNA lesions associated with triplex-forming oligonucleotides. *Mol Carcinog.* (2009) 48:389–99. 10.1002/mc.20501 19072762 PMC2745299

[21] Ricciardi AS McNeer NA Anandalingam KK Saltzman WM Glazer PM. Targeted genome modification via triple helix formation. *Methods Mol Biol.* (2014) 1176:89–106. 10.1007/978-1-4939-0992-6_8 25030921 PMC5111905

[22] McNeer NA Schleifman EB Glazer PM Saltzman WM. Polymer delivery systems for site-specific genome editing. *J Control Release.* (2011) 155:312–6. 10.1016/j.jconrel.2011.05.011 21620910 PMC3176956

[23] Jinek M Jiang F Taylor DW Sternberg SH Kaya E Ma E Structures of Cas9 endonucleases reveal RNA-mediated conformational activation. *Science.* (2014) 343:1247997. 10.1126/science.1247997 24505130 PMC4184034

[24] Nishimasu H Ran FA Hsu PD Konermann S Shehata SI Dohmae N Crystal structure of Cas9 in complex with guide RNA and target DNA. *Cell.* (2014) 156:935–49. 10.1016/j.cell.2014.02.001 24529477 PMC4139937

[25] Rees HA Liu DR. Base editing: precision chemistry on the genome and transcriptome of living cells. *Nat Rev Genet.* (2018) 19:770–88. 10.1038/s41576-018-0059-1 30323312 PMC6535181

[26] Liu X Meng FL. Generation of genomic alteration from cytidine deamination. *Adv Exp Med Biol.* (2018) 1044:49–64. 10.1007/978-981-13-0593-1_5 29956291

[27] Salter JD Bennett RP Smith HC. The APOBEC protein family: united by structure, divergent in function. *Trends Biochem Sci.* (2016) 41:578–94. 10.1016/j.tibs.2016.05.001 27283515 PMC4930407

[28] Komor AC Kim YB Packer MS Zuris JA Liu DR. Programmable editing of a target base in genomic DNA without double-stranded DNA cleavage. *Nature.* (2016) 533:420–4. 10.1038/nature17946 27096365 PMC4873371

[29] Nishida K Arazoe T Yachie N Banno S Kakimoto M Tabata M Targeted nucleotide editing using hybrid prokaryotic and vertebrate adaptive immune systems. *Science.* (2016) 353:aaf8729. 10.1126/science.aaf8729 27492474

[30] Hung KL Meitlis I Hale M Chen CY Singh S Jackson SW Engineering protein-secreting plasma cells by homology-directed repair in primary human B cells. *Mol Ther.* (2018) 26:456–67. 10.1016/j.ymthe.2017.11.012 29273498 PMC5835153

[31] Gaudelli NM Komor AC Rees HA Packer MS Badran AH Bryson DI Programmable base editing of A^∗^T to G^∗^C in genomic DNA without DNA cleavage. *Nature.* (2017) 551:464–71. 10.1038/nature24644 29160308 PMC5726555

[32] Koblan LW Doman JL Wilson C Levy JM Tay T Newby GA Improving cytidine and adenine base editors by expression optimization and ancestral reconstruction. *Nat Biotechnol.* (2018) 36:843–6. 10.1038/nbt.4172 29813047 PMC6126947

[33] Gaudelli NM Lam DK Rees HA Sola-Esteves NM Barrera LA Born DA Directed evolution of adenine base editors with increased activity and therapeutic application. *Nat Biotechnol.* (2020) 38:892–900. 10.1038/s41587-020-0491-6 32284586

[34] Martin F Sanchez-Hernandez S Gutierrez-Guerrero A Pinedo-Gomez J Benabdellah K. Biased and unbiased methods for the detection of off-target cleavage by CRISPR/Cas9: an overview. *Int J Mol Sci.* (2016) 17:1507. 10.3390/ijms17091507 27618019 PMC5037784

[35] Jin S Zong Y Gao Q Zhu Z Wang Y Qin P Cytosine, but not adenine, base editors induce genome-wide off-target mutations in rice. *Science.* (2019) 364:292–5. 10.1126/science.aaw7166 30819931

[36] Zuo E Sun Y Wei W Yuan T Ying W Sun H Cytosine base editor generates substantial off-target single-nucleotide variants in mouse embryos. *Science.* (2019) 364:289–92. 10.1126/science.aav9973 30819928 PMC7301308

[37] Grunewald J Zhou R Garcia SP Iyer S Lareau CA Aryee MJ Transcriptome-wide off-target RNA editing induced by CRISPR-guided DNA base editors. *Nature.* (2019) 569:433–7. 10.1038/s41586-019-1161-z 30995674 PMC6657343

[38] Kleinstiver BP Pattanayak V Prew MS Tsai SQ Nguyen NT Zheng Z High-fidelity CRISPR-Cas9 nucleases with no detectable genome-wide off-target effects. *Nature.* (2016) 529:490–5. 10.1038/nature16526 26735016 PMC4851738

[39] Flotte TR Gao G. Prime editing: a novel Cas9-reverse transcriptase fusion may revolutionize genome editing. *Hum Gene Ther.* (2019) 30:1445–6. 10.1089/hum.2019.29098.trf 31860398

[40] Cohen J. Prime editing promises to be a cut above CRISPR. *Science.* (2019) 366:406. 10.1126/science.366.6464.406 31649173

[41] Anzalone AV Randolph PB Davis JR Sousa AA Koblan LW Levy JM Search-and-replace genome editing without double-strand breaks or donor DNA. *Nature.* (2019) 576:149–57. 10.1038/s41586-019-1711-4 31634902 PMC6907074

[42] Naldini L Trono D Verma IM. Lentiviral vectors, two decades later. *Science.* (2016) 353:1101–2. 10.1126/science.aah6192 27609877

[43] Naldini L. Gene therapy returns to centre stage. *Nature.* (2015) 526:351–60. 10.1038/nature15818 26469046

[44] Sugita M Guzman ML. CD123 as a therapeutic target against malignant stem cells. *Hematol Oncol Clin North Am.* (2020) 34:553–64. 10.1016/j.hoc.2020.01.004 32336419

[45] Stevens BM Zhang W Pollyea DA Winters A Gutman J Smith C CD123 CAR T cells for the treatment of myelodysplastic syndrome. *Exp Hematol.* (2019) 74:52–63e3. 10.1016/j.exphem.2019.05.002 31136781 PMC7184766

[46] Testa U Pelosi E Castelli G. CD123 as a therapeutic target in the treatment of hematological malignancies. *Cancers (Basel).* (2019) 11:1358. 10.3390/cancers11091358 31547472 PMC6769702

[47] Kim MY Yu KR Kenderian SS Ruella M Chen S Shin TH Genetic inactivation of CD33 in hematopoietic stem cells to enable CAR T cell immunotherapy for acute myeloid leukemia. *Cell.* (2018) 173:1439–53.e19. 29856956 10.1016/j.cell.2018.05.013 PMC6003425

[48] Cheong TC Compagno M Chiarle R. Editing of mouse and human immunoglobulin genes by CRISPR-Cas9 system. *Nat Commun.* (2016) 7:10934. 10.1038/ncomms10934 26956543 PMC4786874

[49] Greiner V Bou Puerto R Liu S Herbel C Carmona EM Goldberg MS. CRISPR-mediated editing of the B cell receptor in primary human B cells. *iScience.* (2019) 12:369–78. 10.1016/j.isci.2019.01.032 30769282 PMC6374785

[50] Voss JE Gonzalez-Martin A Andrabi R Fuller RP Murrell B McCoy LE Reprogramming the antigen specificity of B cells using genome-editing technologies. *eLife.* (2019) 8:e42995. 10.7554/eLife.42995 30648968 PMC6355199

[51] Hartweger H McGuire AT Horning M Taylor JJ Dosenovic P Yost D HIV-specific humoral immune responses by CRISPR/Cas9-edited B cells. *J Exp Med.* (2019) 216:1301–10. 10.1084/jem.20190287 30975893 PMC6547862

[52] Beane JD Lee G Zheng Z Mendel M Abate-Daga D Bharathan M Clinical scale zinc finger nuclease-mediated gene editing of PD-1 in tumor infiltrating lymphocytes for the treatment of metastatic melanoma. *Mol Ther.* (2015) 23:1380–90. 10.1038/mt.2015.71 25939491 PMC4817870

[53] Menger L Sledzinska A Bergerhoff K Vargas FA Smith J Poirot L TALEN-mediated inactivation of PD-1 in tumor-reactive lymphocytes promotes intratumoral T-cell persistence and rejection of established tumors. *Cancer Res.* (2016) 76:2087–93. 10.1158/0008-5472.CAN-15-3352 27197251

[54] Choi BD Yu X Castano AP Darr H Henderson DB Bouffard AA CRISPR-Cas9 disruption of PD-1 enhances activity of universal EGFRvIII CAR T cells in a preclinical model of human glioblastoma. *J Immunother Cancer.* (2019) 7:304. 10.1186/s40425-019-0806-7 31727131 PMC6857271

[55] Webber BR Lonetree CL Kluesner MG Johnson MJ Pomeroy EJ Diers MD Highly efficient multiplex human T cell engineering without double-strand breaks using Cas9 base editors. *Nat Commun.* (2019) 10:5222. 10.1038/s41467-019-13778-y 31745080 PMC6864045

[56] Torikai H Reik A Liu PQ Zhou Y Zhang L Maiti S A foundation for universal T-cell based immunotherapy: T cells engineered to express a CD19-specific chimeric-antigen-receptor and eliminate expression of endogenous TCR. *Blood.* (2012) 119:5697–705. 10.1182/blood-2012-01-405365 22535661 PMC3382929

[57] Ren J Liu X Fang C Jiang S June CH Zhao Y. Multiplex genome editing to generate universal CAR T cells resistant to PD1 inhibition. *Clin Cancer Res.* (2017) 23:2255–66. 10.1158/1078-0432.CCR-16-1300 27815355 PMC5413401

[58] Ren J Zhang X Liu X Fang C Jiang S June CH A versatile system for rapid multiplex genome-edited CAR T cell generation. *Oncotarget.* (2017) 8:17002–11. 10.18632/oncotarget.15218 28199983 PMC5370017

[59] Gornalusse GG Hirata RK Funk SE Riolobos L Lopes VS Manske G HLA-E-expressing pluripotent stem cells escape allogeneic responses and lysis by NK cells. *Nat Biotechnol.* (2017) 35:765–72. 10.1038/nbt.3860 28504668 PMC5548598

[60] Riolobos L Hirata RK Turtle CJ Wang PR Gornalusse GG Zavajlevski M HLA engineering of human pluripotent stem cells. *Mol Ther.* (2013) 21:1232–41. 10.1038/mt.2013.59 23629003 PMC3677304

[61] Berdien B Mock U Atanackovic D Fehse B. TALEN-mediated editing of endogenous T-cell receptors facilitates efficient reprogramming of T lymphocytes by lentiviral gene transfer. *Gene Ther.* (2014) 21:539–48. 10.1038/gt.2014.26 24670996

[62] Seki A Rutz S. Optimized RNP transfection for highly efficient CRISPR/Cas9-mediated gene knockout in primary T cells. *J Exp Med.* (2018) 215:985–97. 10.1084/jem.20171626 29436394 PMC5839763

[63] Legut M Dolton G Mian AA Ottmann OG Sewell AK. CRISPR-mediated TCR replacement generates superior anticancer transgenic T cells. *Blood.* (2018) 131:311–22. 10.1182/blood-2017-05-787598 29122757 PMC5774207

[64] Osborn MJ Webber BR Knipping F Lonetree CL Tennis N DeFeo AP Evaluation of TCR gene editing achieved by TALENs, CRISPR/Cas9, and megaTAL nucleases. *Mol Ther.* (2016) 24:570–81. 10.1038/mt.2015.197 26502778 PMC4786913

[65] Tang N Cheng C Zhang X Qiao M Li N Mu W TGF-beta inhibition via CRISPR promotes the long-term efficacy of CAR T cells against solid tumors. *JCI Insight.* (2020) 5:e133977. 10.1172/jci.insight.133977 31999649 PMC7101140

[66] Kok VC. Current understanding of the mechanisms underlying immune evasion from PD-1/PD-L1 immune checkpoint blockade in head and neck cancer. *Front Oncol.* (2020) 10:268. 10.3389/fonc.2020.00268 32185135 PMC7058818

[67] Teh JLF Aplin AE. Arrested developments: CDK4/6 inhibitor resistance and alterations in the tumor immune microenvironment. *Clin Cancer Res.* (2019) 25:921–7. 10.1158/1078-0432.CCR-18-1967 30287548 PMC6359975

[68] Dorand RD Nthale J Myers JT Barkauskas DS Avril S Chirieleison SM Cdk5 disruption attenuates tumor PD-L1 expression and promotes antitumor immunity. *Science.* (2016) 353:399–403. 10.1126/science.aae0477 27463676 PMC5051664

[69] Ardelt MA Frohlich T Martini E Muller M Kanitz V Atzberger C Inhibition of cyclin-dependent kinase 5: a strategy to improve sorafenib response in hepatocellular carcinoma therapy. *Hepatology.* (2019) 69:376–93. 10.1002/hep.30190 30033593 PMC6590289

[70] Gao L Shay C Lv F Wang X Teng Y. Implications of FGF19 on sorafenib-mediated nitric oxide production in hepatocellular carcinoma cells – a short report. *Cell Oncol (Dordr).* (2018) 41:85–91. 10.1007/s13402-017-0354-4 28983785 PMC12995209

[71] Stadtmauer EA Fraietta JA Davis MM Cohen AD Weber KL Lancaster E CRISPR-engineered T cells in patients with refractory cancer. *Science.* (2020) 367:eaba7365. 10.1126/science.aba7365 32029687 PMC11249135

[72] Poirot L Philip B Schiffer-Mannioui C Le Clerre D Chion-Sotinel I Derniame S Multiplex genome-edited T-cell manufacturing platform for “Off-the-Shelf” adoptive T-cell immunotherapies. *Cancer Res.* (2015) 75:3853–64. 10.1158/0008-5472.CAN-14-3321 26183927

[73] Qasim W Zhan H Samarasinghe S Adams S Amrolia P Stafford S Molecular remission of infant B-ALL after infusion of universal TALEN gene-edited CAR T cells. *Sci Transl Med.* (2017) 9:eaaj2013. 10.1126/scitranslmed.aaj2013 28123068

[74] Gautron AS Juillerat A Guyot V Filhol JM Dessez E Duclert A Fine and predictable tuning of TALEN gene editing targeting for improved T cell adoptive immunotherapy. *Mol Ther Nucleic Acids.* (2017) 9:312–21. 10.1016/j.omtn.2017.10.005 29246309 PMC5684446

[75] Roth TL Puig-Saus C Yu R Shifrut E Carnevale J Li PJ Reprogramming human T cell function and specificity with non-viral genome targeting. *Nature.* (2018) 559:405–9. 10.1038/s41586-018-0326-5 29995861 PMC6239417

[76] Eyquem J Mansilla-Soto J Giavridis T van der Stegen SJ Hamieh M Cunanan KM Targeting a CAR to the TRAC locus with CRISPR/Cas9 enhances tumour rejection. *Nature.* (2017) 543:113–7. 10.1038/nature21405 28225754 PMC5558614

[77] Sachdeva M Busser BW Temburni S Jahangiri B Gautron AS Marechal A Repurposing endogenous immune pathways to tailor and control chimeric antigen receptor T cell functionality. *Nat Commun.* (2019) 10:5100. 10.1038/s41467-019-13088-3 31723132 PMC6853973

[78] Johansson S Berg L Hall H Hoglund P. NK cells: elusive players in autoimmunity. *Trends Immunol.* (2005) 26:613–8. 10.1016/j.it.2005.08.008 16118064

[79] Pfefferle A Jacobs B Haroun-Izquierdo A Kveberg L Sohlberg E Malmberg KJ. Deciphering natural killer cell homeostasis. *Front Immunol.* (2020) 11:812. 10.3389/fimmu.2020.00812 32477340 PMC7235169

[80] Gang M Marin ND Wong P Neal CC Marsala L Foster M CAR-modified memory-like NK cells exhibit potent responses to NK-resistant lymphomas. *Blood.* (2020): 10.1182/blood.2020006619 [Epub ahead of print]. 32614951 PMC7702478

[81] Siegler EL Zhu Y Wang P Yang L. Off-the-shelf CAR-NK cells for cancer immunotherapy. *Cell Stem Cell.* (2018) 23:160–1. 10.1016/j.stem.2018.07.007 30075127

[82] Pinz KG Yakaboski E Jares A Liu H Firor AE Chen KH Targeting T-cell malignancies using anti-CD4 CAR NK-92 cells. *Oncotarget.* (2017) 8:112783–96. 10.18632/oncotarget.22626 29348865 PMC5762550

[83] Naeimi Kararoudi M Hejazi SS Elmas E Hellstrom M Padma AM Lee D Clustered regularly interspaced short palindromic repeats/Cas9 gene editing technique in xenotransplantation. *Front Immunol.* (2018) 9:1711. 10.3389/fimmu.2018.01711 30233563 PMC6134075

[84] Pomeroy EJ Hunzeker JT Kluesner MG Lahr WS Smeester BA Crosby MR A genetically engineered primary human natural killer cell platform for cancer immunotherapy. *Mol Ther.* (2020) 28:52–63. 10.1016/j.ymthe.2019.10.009 31704085 PMC6953961

[85] Mishra HK Pore N Michelotti EF Walcheck B. Anti-ADAM17 monoclonal antibody MEDI3622 increases IFNgamma production by human NK cells in the presence of antibody-bound tumor cells. *Cancer Immunol Immunother.* (2018) 67:1407–16. 10.1007/s00262-018-2193-1 29978334 PMC6126979

[86] Bourque J Hawiger D. Immunomodulatory bonds of the partnership between dendritic cells and T cells. *Crit Rev Immunol.* (2018) 38:379–401. 10.1615/CritRevImmunol.2018026790 30792568 PMC6380512

[87] Ara A Ahmed KA Xiang J. Multiple effects of CD40-CD40L axis in immunity against infection and cancer. *Immunotargets Ther.* (2018) 7:55–61. 10.2147/ITT.S163614 29988701 PMC6029590

[88] Zhang Y Shen S Zhao G Xu CF Zhang HB Luo YL In situ repurposing of dendritic cells with CRISPR/Cas9-based nanomedicine to induce transplant tolerance. *Biomaterials.* (2019) 217:119302. 10.1016/j.biomaterials.2019.119302 31271858

[89] Luo YL Xu CF Li HJ Cao ZT Liu J Wang JL Macrophage-specific in vivo gene editing using cationic lipid-assisted polymeric nanoparticles. *ACS Nano.* (2018) 12:994–1005. 10.1021/acsnano.7b07874 29314827

[90] Ray M Lee YW Hardie J Mout R Yesilbag Tonga G Farkas ME CRISPRed macrophages for cell-based cancer immunotherapy. *Bioconjug Chem.* (2018) 29:445–50. 10.1021/acs.bioconjchem.7b00768 29298051 PMC6063311

[91] Sommer C Boldajipour B Kuo TC Bentley T Sutton J Chen A Preclinical evaluation of allogeneic CAR T cells targeting BCMA for the treatment of multiple myeloma. *Mol Ther.* (2019) 27:1126–38. 10.1016/j.ymthe.2019.04.001 31005597 PMC6554542

[92] Liu X Zhang Y Cheng C Cheng AW Zhang X Li N CRISPR-Cas9-mediated multiplex gene editing in CAR-T cells. *Cell Res.* (2017) 27:154–7. 10.1038/cr.2016.142 27910851 PMC5223227

[93] Rasaiyaah J Georgiadis C Preece R Mock U Qasim W. TCRalphabeta/CD3 disruption enables CD3-specific antileukemic T cell immunotherapy. *JCI Insight.* (2018) 3:e99442. 10.1172/jci.insight.99442 29997304 PMC6124532

[94] Mamonkin M Mukherjee M Srinivasan M Sharma S Gomes-Silva D Mo F Reversible transgene expression reduces fratricide and permits 4-1BB costimulation of CAR T cells directed to T-cell malignancies. *Cancer Immunol Res.* (2018) 6:47–58. 10.1158/2326-6066.CIR-17-0126 29079655 PMC5963729

[95] Gomes-Silva D Srinivasan M Sharma S Lee CM Wagner DL Davis TH CD7-edited T cells expressing a CD7-specific CAR for the therapy of T-cell malignancies. *Blood.* (2017) 130:285–96. 10.1182/blood-2017-01-761320 28539325 PMC5520470

[96] Cooper ML Choi J Staser K Ritchey JK Devenport JM Eckardt K An “off-the-shelf” fratricide-resistant CAR-T for the treatment of T cell hematologic malignancies. *Leukemia.* (2018) 32:1970–83. 10.1038/s41375-018-0065-5 29483708 PMC6102094

[97] Cheng X Veverka V Radhakrishnan A Waters LC Muskett FW Morgan SH Structure and interactions of the human programmed cell death 1 receptor. *J Biol Chem.* (2013) 288:11771–85. 10.1074/jbc.M112.448126 23417675 PMC3636866

[98] Lin G Chen S Mi P. Nanoparticles targeting and remodeling tumor microenvironment for cancer theranostics. *J Biomed Nanotechnol.* (2018) 14:1189–207. 10.1166/jbn.2018.2546 29944095

[99] Sunshine J Taube JM. PD-1/PD-L1 inhibitors. *Curr Opin Pharmacol.* (2015) 23:32–8. 10.1016/j.coph.2015.05.011 26047524 PMC4516625

[100] Guo L Zhang H Chen B. Nivolumab as programmed death-1 (PD-1) inhibitor for targeted immunotherapy in tumor. *J Cancer.* (2017) 8:410–6. 10.7150/jca.17144 28261342 PMC5332892

[101] Powles T Eder JP Fine GD Braiteh FS Loriot Y Cruz C MPDL3280A (anti-PD-L1) treatment leads to clinical activity in metastatic bladder cancer. *Nature.* (2014) 515:558–62. 10.1038/nature13904 25428503

[102] Lu S Yang N He J Gong W Lai Z Xie L Generation of cancer-specific cytotoxic PD-1(-) T cells using liposome-encapsulated CRISPR/Cas system with dendritic/tumor fusion cells. *J Biomed Nanotechnol.* (2019) 15:593–601. 10.1166/jbn.2019.2712 31165703

[103] Su S Hu B Shao J Shen B Du J Du Y CRISPR-Cas9 mediated efficient PD-1 disruption on human primary T cells from cancer patients. *Sci Rep.* (2016) 6:20070. 10.1038/srep20070 26818188 PMC4730182

[104] Chen M Xu J Zhou Y Zhang S Zhu D. CRISPR-Cas9 genome editing for cancer immunotherapy: opportunities and challenges. *Brief Funct Genomics.* (2020) 19:183–90. 10.1093/bfgp/elz027 31788683

[105] Beavis PA Henderson MA Giuffrida L Mills JK Sek K Cross RS Targeting the adenosine 2A receptor enhances chimeric antigen receptor T cell efficacy. *J Clin Invest.* (2017) 127:929–41. 10.1172/JCI89455 28165340 PMC5330718

[106] Shi L Meng T Zhao Z Han J Zhang W Gao F CRISPR knock out CTLA-4 enhances the anti-tumor activity of cytotoxic T lymphocytes. *Gene.* (2017) 636:36–41. 10.1016/j.gene.2017.09.010 28888577

[107] Zhang W Shi L Zhao Z Du P Ye X Li D Disruption of CTLA-4 expression on peripheral blood CD8 + T cell enhances anti-tumor efficacy in bladder cancer. *Cancer Chemother Pharmacol.* (2019) 83:911–20. 10.1007/s00280-019-03800-x 30848330

[108] Nguyen LT Ohashi PS. Clinical blockade of PD1 and LAG3–potential mechanisms of action. *Nat Rev Immunol.* (2015) 15:45–56. 10.1038/nri3790 25534622

[109] Anderson AC Joller N Kuchroo VK. Lag-3, Tim-3, and TIGIT: co-inhibitory receptors with specialized functions in immune regulation. *Immunity.* (2016) 44:989–1004. 10.1016/j.immuni.2016.05.001 27192565 PMC4942846

[110] Benjamin R Berges BK Solis-Leal A Igbinedion O Strong CL Schiller MR. TALEN gene editing takes aim on HIV. *Hum Genet.* (2016) 135:1059–70. 10.1007/s00439-016-1678-2 27170155 PMC5002248

[111] Cong L Ran FA Cox D Lin S Barretto R Habib N Multiplex genome engineering using CRISPR/Cas systems. *Science.* (2013) 339:819–23. 10.1126/science.1231143 23287718 PMC3795411

[112] Fraietta JA Lacey SF Orlando EJ Pruteanu-Malinici I Gohil M Lundh S Determinants of response and resistance to CD19 chimeric antigen receptor (CAR) T cell therapy of chronic lymphocytic leukemia. *Nat Med.* (2018) 24:563–71. 10.1038/s41591-018-0010-1 29713085 PMC6117613

[113] Zhang Q Chikina M Szymczak-Workman AL Horne W Kolls JK Vignali KM LAG3 limits regulatory T cell proliferation and function in autoimmune diabetes. *Sci Immunol.* (2017) 2:eaah4569. 10.1126/sciimmunol.aah4569 28783703 PMC5609824

[114] Delacote F Perez C Guyot V Mikonio C Potrel P Cabaniols JP Identification of genes regulating gene targeting by a high-throughput screening approach. *J Nucleic Acids.* (2011) 2011:947212. 10.4061/2011/947212 21716659 PMC3118287

[115] Beard WA Horton JK Prasad R Wilson SH. Eukaryotic base excision repair: new approaches shine light on mechanism. *Annu Rev Biochem.* (2019) 88:137–62. 10.1146/annurev-biochem-013118-111315 31220977 PMC8956022

[116] Komor AC Zhao KT Packer MS Gaudelli NM Waterbury AL Koblan LW Improved base excision repair inhibition and bacteriophage Mu Gam protein yields C:G-to-T:A base editors with higher efficiency and product purity. *Sci Adv.* (2017) 3:eaao4774. 10.1126/sciadv.aao4774 28875174 PMC5576876

[117] Naeimi Kararoudi M Dolatshad H Trikha P Hussain SA Elmas E Foltz JA Generation of knock-out primary and expanded human nk cells using Cas9 ribonucleoproteins. *J Vis Exp.* (2018) 136:58237. 10.3791/58237 29985369 PMC6101749

[118] Freund EC Lock JY Oh J Maculins T Delamarre L Bohlen CJ Efficient gene knockout in primary human and murine myeloid cells by non-viral delivery of CRISPR-Cas9. *J Exp Med.* (2020) 217:e20191692. 10.1084/jem.20191692 32357367 PMC7336301

[119] Palazon-Riquelme P Worboys JD Green J Valera A Martin-Sanchez F Pellegrini C USP7 and USP47 deubiquitinases regulate NLRP3 inflammasome activation. *EMBO Rep.* (2018) 19:e44766. 10.15252/embr.201744766 30206189 PMC6172458

[120] Sieve I Ricke-Hoch M Kasten M Battmer K Stapel B Falk CS A positive feedback loop between IL-1beta, LPS and NEU1 may promote atherosclerosis by enhancing a pro-inflammatory state in monocytes and macrophages. *Vascul Pharmacol.* (2018) 103-105:16–28. 10.1016/j.vph.2018.01.005 29371126

